# Correspondence

**Published:** 1877-11

**Authors:** R. M. Lackey

**Affiliations:** Boston


### Correspondence.

BOSTON, September 26, 1877.

EDS. MEDICAL JOURNAL AND EXAMINER:—Boston is a place of great pretensions, and one naturally comes here with great expectations. No one need be disappointed either, unless he entertains unreasonably extravagant notions of the "Hub" and its institutions. Her medical men and medical institutions are inferior to none in the country. Her institutions, in all departments, show the perfection that comes with age, and capable management. Nowhere in the country, perhaps, is there so large a proportion of physicians who are gentlemen of superior education and literary and scientific culture as here; among the young men too who are preparing to enter the profession, a very large proportion have received a thorough academic training; and the mental discipline thus obtained will tell in their whole career. Unlike other cities of this country, Boston is not overstocked with medical colleges, the Harvard Medical School being the only regular one, and besides this is the medical department of Boston University, a small institution which deals out only small pills.

Now I cannot continue to say only *good* things of this city, for in spite of the numbers and culture and ability of the regular profession, the place certainly takes the palm for quacks, charlatans, humbugs and "isms" of various kinds. I counted in one of the daily papers the other morning, fifteen quack doctors' advertisements, occupying nearly the whole of one side of the paper; and in addition to these, thirty cards of clairvoyants, magnetic doctors, etc. Homeopathy, too, has its adherents, as has the "faith cure," which might perhaps be appropriately classed with the homeopathic system, as faith is the essential in both. While dwelling on the "faith cure," I will mention a case I saw at "The Consumptive's Home,"—

an institution which claims to receive its support and healing through "faith,"—of a paraplegic woman who has been "trusting" for the healing power these seven years, and has refused all medication or other curative means. Aphonia has existed in this case for twenty years, and even this feature of her affliction has received no benefit from this prodigious exercise of faith. While at this institution, I asked for the prevailing treatment of the consumptives, and the reply was, that some were treated homeopathically and others took no medicine or special nutrition, but trusted to the prayers of faith. "And what percentage of your patients are discharged cured," I asked. "Oh, very few, the cases most all die," was the reply, "whether they take medicine or trust wholly to supernatural power."

The city hospital is the great municipal medical charity of this city. It is admirably located for healthfulness and convenience; the grounds, about seven acres, are large, and near the salt water; the buildings are ample and mainly of the most approved model. It was one of the first erected on the pavilion plan; the first pavilions erected are somewhat defective in ventilation, but the last put up are perfect in this regard, having ridge ventilation.

The superiority of this kind of hospital ward, and this system of ventilation are so evident, that it is a wonder to me, as it is to the medical gentlemen here who have such a good chance for comparison, that at this day any other kind should be erected, especially in cities like Chicago where space is so abundant and cheap. I was pleased with the care exercised by the internes of this hospital in diagnosis, and the simplicity and judiciousness of the treatment. The expectant treatment, and the judicious, cautious use of medicines, advocated by the late Dr. Jackson, seem to have taken root here. The tub bath is largely used in the treatment of typhoid fever, and with satisfactory results. The patient is placed in an ordinary bath tub, with the water at a temperature of 80 or 90 degrees (F.), and gradually cooled by the addition of cold water. Of thirty-two cases reported treated by this method, only one died. A case I saw in one of the wards was treated during the stage of exhaustion by hypordermic injections of brandy, and with some apparent benefit. Salicylic acid is used for rheumatism, in large doses frequently repeated for a day or two, and with good results. Jaborandi has been on trial here, but has not sustained the reputation given it by some.

On the surgical side the material for clinical study is abundant. I notice that in fractures of the leg, especially compound, the good old fracture box is still used, and for the thigh, extension by weight and pulley, and short splints are used, and the results are most satisfactory. I think that here less weight is used than is customary by some surgeons I know, and less than in some other hospitals I have visited.

The antiseptic method has been tested in this institution, but now rather fallen into disuse, as the results were not, in the hands of the surgeons here, those claimed for it by Lister and others. Dr. Blake reports 19 cases of empyema treated by permanent openings of the thoracic cavity; fifteen of the number were cured, or greatly benefited by this method of treatment. I was shown in the children's ward a case of syphilis in a little girl 10 years old; she had a large chancre on one of the labia, and inflammation of the inguinal glands. The disease in this case, too, was communicated in the ordinary way.

In my visits to the medical institutions I have not slighted the Charitable Eye and Ear Infirmary. It is, I believe, a kind of mother to similar institutions of the kind throughout the country; and yet it shows no signs of decrepitude or failing usefulness. It is admirably conducted, and furnishes an exhaustless amount of material for clinical instruction in eye and ear diseases; the instructors are among the most eminent in the country in their specialty.

The abuse of medical charities by those able to pay for medical attendance is receiving the attention of the profession here, as it is in nearly all the large cities in this country and Europe.

At the eye and ear infirmary I observed a large proportion of the patients were well-dressed and well-fed; and Dr. Jeffries made the remark that "these people often come in a hurry with a story of a baby at home, etc., when really a carriage was waiting to take them to an excursion boat or train, or a-shopping." How to protect charitable institutions from such shameful lying and deception, is a matter of serious import.

My letter is getting long, and I would stop, were I not so anxious to give your readers a brief abstract of a case I saw operated upon by Dr. J. C. Warren, in the Mass. General Hospital, to-day.

The patient was a young woman aged 21. At the age of 16, she met with the loss of her entire scalp, including the eyebrows and a part of the skin of the right cheek. While at work in a factory, her hair was caught by a belt and shaft and the scalp torn away in an instant. Hemorrhage was slight; the scalp was picked up, replaced and stitched on, but it dried up and was clipped away entirely. She was removed at once after the injury to the Massachusetts General Hospital, and remained under Dr. Warren's care for eight weeks. Several attempts at skin-grafting were made, but unsuccessfully. She then returned to her home at Stoneham, Mass., and came under the care of Dr. W. Symington Brown. Dr. B. used a weak solution of carbolic acid locally at first, and afterwards a weak solution of bromine in sol. of bromide of potass. In about 8 months from the receipt of the injury, Dr. B. commenced skin-grafting once a week, and continued it for three years. During that time 2,600 pieces of skin were applied; the grafts being furnished by 180 different persons; mostly young people and children; one girl furnishing 103 grafts.

Since the accident she has had three attacks of pyemia; the last one accompanied by the appearance of petechial spots over the whole body, and partial suppression of urine. The entire scalp is now healed over, except a small red smooth spot above the left eye. This is a portion of the conjunctiva which was torn from its normal position and reflected upon the eyebrow, and which closely resembles an ordinary granulating wound; but after faithful trial the skin grafts would not take here as on the rest of the scalp, and to-day Dr. Warren performed an operation with a view to replacing the conjunctiva by dissecting it from its attachment to the eyebrow, and stitching the free border to the eyelid. The raw surface was then covered with a piece of skin as large as a half-dollar taken from the patient's thigh. Large skin-grafts have succeeded in a few cases reported; but in this case nothing was claimed more than an experiment. If the large graft failed, the surface could afterwards be treated with the smaller ones, as was done with the rest of the scalp. The eyes were not injured further than the laceration of the lids in consequence of which the left eye is partially closed. The vision is so good that she does sewing and takes lessons in music. After the scalp began to heal, the downy hair on the neck took on an extraordinary growth, and this hair is now fourteen inches long and curly. I propose to follow up this case and learn, if possible, the result of Dr. Warren's operation to-day, and may give your readers the benefit of it. The case is one of great interest, in showing the value of skin-grafting when followed up with the commendable perseverance exhibited by the gentlemen who had charge of this case. R. M. LACKEY.

